# Nanomedicines for the management of diabetic nephropathy: present progress and prospects

**DOI:** 10.3389/fendo.2023.1236686

**Published:** 2023-11-03

**Authors:** Paramita Paul, Leena Chacko, Tarun K. Dua, Pratik Chakraborty, Udita Paul, Vishwakarma Vishal Phulchand, Niraj K. Jha, Saurabh K. Jha, Ramesh Kandimalla, Saikat Dewanjee

**Affiliations:** ^1^ Department of Pharmaceutical Technology, University of North Bengal, Darjeeling, India; ^2^ BioAnalytical Lab, Meso Scale Discovery, Rockville, MD, United States; ^3^ Advanced Pharmacognosy Research Laboratory, Department of Pharmaceutical Technology, Jadavpur University, Kolkata, India; ^4^ Department of Biotechnology, School of Engineering & Technology, Sharda University, Greater Noida, Uttar Pradesh, India; ^5^ Department of Biochemistry, Kakatiya Medical College, Warangal, Telangana, India; ^6^ Department of Applied Biology, Indian Institute of Technology, Council of Scientific & Industrial Research (CSIR), Hyderabad, India

**Keywords:** diabetic nephropathy, glomerular filtration barrier, nanocarriers, nanotheranostics, renal retention, targeted delivery

## Abstract

Diabetic nephropathy (DN) is a serious microvascular consequence of diabetes mellitus (DM), posing an encumbrance to public health worldwide. Control over the onset and progress of DN depend heavily on early detection and effective treatment. DN is a major contributor to end-stage renal disease, and a complete cure is yet to be achieved with currently available options. Though some therapeutic molecules have exhibited promise in treating DN complications, their poor solubility profile, low bioavailability, poor permeation, high therapeutic dose and associated toxicity, and low patient compliance apprehend their clinical usefulness. Recent research has indicated nano-systems as potential theranostic platforms displaying futuristic promise in the diagnosis and treatment of DN. Early and accurate diagnosis, site-specific delivery and retention by virtue of ligand conjugation, and improved pharmacokinetic profile are amongst the major advantages of nano-platforms, defining their superiority. Thus, the emergence of nanoparticles has offered fresh approaches to the possible diagnostic and therapeutic strategies regarding DN. The present review corroborates an updated overview of different types of nanocarriers regarding potential approaches for the diagnosis and therapy of DN.

## 1 Introduction

Diabetes mellitus (DM) is linked to long-term damage and failure of various organs to function correctly and is a major contributor to the onset of many associated complications (1, 2). Diabetic nephropathy (DN) is one of the most frequent and severe forms of diabetic complications linked to diabetes-related microvascular damage, renal failure, and overt albuminuria. In most cases, renal complications are accompanied by cardiovascular irregularities contributing to a shortened lifespan of the patients (3). DN affects nearly one-third of DM patients, and its prevalence is increasing alarmingly with time (4). As the number of people with DM increases, so does the number of people with DN. DN is also the major cause of end-stage renal diseases, and diabetes-related mortality worldwide (5, 6). Nephromegaly and a modified Doppler are early morphological markers of renal injury, although proteinuria and glomerular filtration rate are the greatest indicators of severity (7). DN is often accompanied by other diabetic microvascular complications like diabetic retinopathy and diabetic neuropathy, which further worsen the situation (8). In addition to cumbersome pathophysiological aspects, DN simultaneously causes financial draining,

Nanoparticles are small nano-sized particles with unique physical, chemical, and biological properties that make them useful as diagnostic and therapeutic tools (9). Nanoparticles have exhibited promising potential regarding the management of DN due to their targeting ability along with improved pharmacokinetic attributes (10). Several nanoformulations have come up with exciting promises in the diagnosis and treatment of DN. Nanoparticles offer several advantages like improvement of pharmacokinetic profiles, stability, bioavailability, trackability, biocompatibility, and therapeutic efficacy of potent chemotherapeutic agents (11). The current article aims to articulate the role of different nanotheranostic tools with utilization promises for the treatment of DN. In this review, present-day advances in the synthesis of DN-selective nanotherapeutics as well as their areas of application have been discussed. The impact of various formulation factors has also been discussed, along with a summary of recent nanotheranostic developments including the passive and active targeting strategies pertinent to DN. The article also discusses present day challenges and/or limitations regarding renal targeting for DN, and attempts to dig out probable remedies for the same. Consequently, prospects of nanomedicine regarding DN management with respect to probable clinical translation has also been discussed in a comprehensive manner.

## 2 DN at a glance

Among diabetic patients, DN is possibly the most life-threatning complication, and leading cause behind renal failure (12). DN is a chronic complication arising from DM over a span of few years (13). The global prevalence of diabetes has risen substantially from 108 million in 1980 to 537 million in 2021, and predicted to rise to 643 million by 2030 and 783 million by 2045 (14). DM-associated hyperglycemia slowly causes hypertension and kidney dysfunction. The developed hypertension further worsens kidney’s functionality, and ultimately results in renal failure. Albuminuria, glomerular lesions, tubulointerstitial fibrosis, and decreased renal filtration rate are the hallmarks of the multifunctional degenerative condition known as DN. Patients with type I DM seldom develop diabetic kidney damage prior to 10 years of the disease, whereas nearly 3% of people with type II DM already have overt nephropathy at the time of first diagnosis (15).

Class I glomerular lesions involve thickening of glomerular basement membrane mainly due to accumulation of extracellular matrix components e.g. collagen IV, laminin, and fibronectin membrane as an early indication of kidney damage. Class II glomerular lesions comprise of mesangial expansions of mild (IIa) to severe (IIb) grades distorting glomerular capillaries. Class III glomerular lesions include mesangiolysis, and detachment of endothelial cells from glomerular basement membrane. Class IV glomerular lesions involve advanced diabetic glomerulosclerosis caused by excessive accumulation of extracellular matrix components. Tubulointerstitial lesions comprise of interstitial fibrosis and tubular atrophy, and interstitial inflammation. Vascular lesions distinguish DN from hypertensive nephropathy. In addition, insudative lesions can be located in Bowman’s capsule, glomerular capillary, and glomerulotubular junction (16). Measures currently used to determine the existence and progression of diabetic nephropathy include blood urea nitrogen, serum creatinine, formulae to estimate glomerular filtration rate, proteinuria, and albuminuria. These measurements, however, are not exact, do not directly assess renal tissue damage, and are comparatively insensitive to minute variations in renal functions. Consequently, the availability of novel biomarkers that are sensitive, specific, and accurate as well as capable of detecting kidney damage and forecasting clinically relevant outcomes would be greatly beneficial in DN.

**Figure 1:**
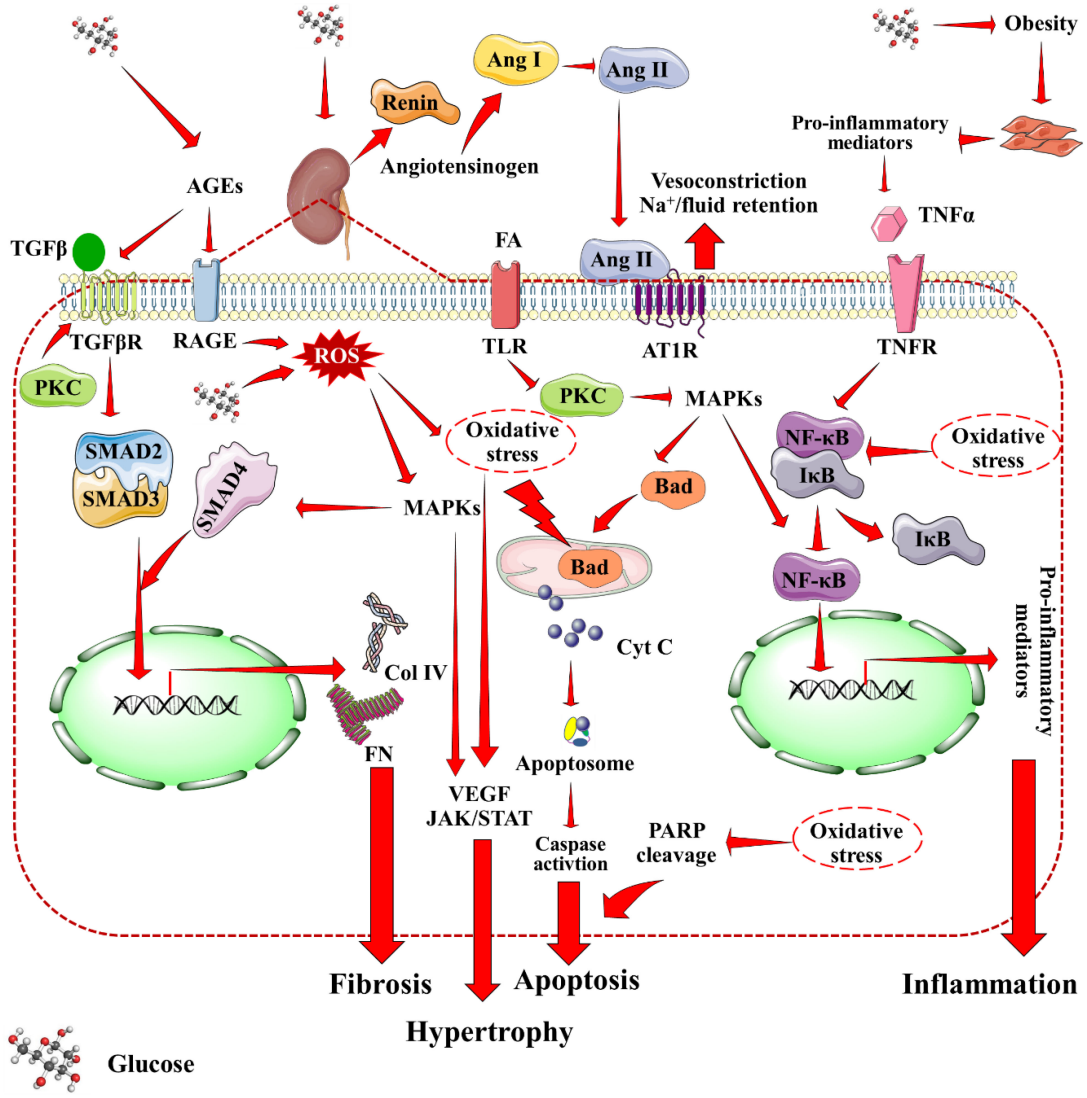
Mechanistic insights into DN-associated signaling cascades. Red arrow indicates downstream cellular events. AGEs, Advanced glycation end products; Ang I, Angiotensin I; Ang II, Angiotensin II; AT1R, Angiotensin II receptor type I; Col IV, Collagen type IV; Cyt C, Cytochrome C; MAPKs, Mitogen activated protein kinases; PARP, Poly (ADP-ribose) polymerase, PKC, Protein kinase C; RAGE, Receptor for AGEs; ROS, Reactive oxygen species; TNFR, Tumor necrosis factor receptor; VEGF, Vascular endothelial growth factor.

The three layer filtration system within the Malpighian corpuscle consists of vascular endothelium, glomerular basement membrane, and podocytes of the visceral epithelium. Mesangial cells produce a collagen network that structurally supports the capillaries. Filtration occurs within glomerulus across mesangium. The primary contributors to the development of DN have been identified as hyperglycemia, overproduction of advanced glycation end products (AGEs), activation of protein kinase C (PKC), increased oxidative stress, inflammation, and activation of poly (ADP-ribose) polymerase (PARP) (12, 17, 18). **Figure 1** provides a holistic overview of DN-associated signaling cascades. An increased pressure state within the nephron is the first development towards DN. DM-associated hypertension results in increased pressure throughout arteriolar-vascular system, including the afferent arteriole of glomerulus. This, in turn increases the glomerular filtration rate. Hyperglycemia-mediated direct intrarenal activation of the renin-angiotensin-aldosterone system (RAAS), and subsequent vasoconstriction of the efferent arteriole further adds to the increased pressure state. Angiotensin II, binding with AT1R receptors causes the smooth muscles of the arteries to contract, raising blood pressure. Additionally the adrenal cortex produces more aldosterone, and sodium absorption is enhanced. During DM associated hypertension, vasoconstriction of efferent artery of glomerulus is greater than that of afferent artery (19). Rise of pressure within the glomerulus results in mesangial expansion. The increased pressure leads to trauma and damage to the mesangium. In response to this damage, mesangial cells start secreting proinflammatory cytokines, and oxygen free radicals that lead to inflammation, oxidative stress, and endothelial dysfunction further damaging the nephron vasculature (20). CD80 upregulation enhances inflammatory cytokine production and mortality. Concurrently, vascular permeability is worsened by AGEs and PKC, which harms the basal membrane. Oxidative stress acts as the trigger for many signaling pathways associated with DN that causes activation of inflammatory, apoptotic and fibrotic pathways especially through the activation of nuclear factor kappa-light-chain-enhancer of activated B cells (NF-κB), mitogen-activated protein kinase (MAPK), mammalian target of rapamycin (mTOR), janus kinase/signal transducers and activators of transcription (JAK/STAT), and (transforming growth factor beta/suppressor of mothers against decapentaplegic) TGF-β/Smad signaling (21, 22). All of these somewhat combine into hypertrophy and matrix accumulation within mesangium, which is known as mesangial expansion (23). TGF-β1, in particular, is linked to glomerular hypertrophy and extracellular matrix accumulation in the mesangium, which reduces glomerular filtration rate and contributes to renal impairment. TGF-β2 overexpression in diabetic kidneys has been linked to renal fibrosis and extracellular matrix accumulation during early stages of DN. Interestingly, hyperglycemia enhances the interaction of dipeptidyl peptidase-4 (DPP-4) with cation-independent mannose 6-phosphate receptors, which results in TGF-β activation in turn leading to renal fibrosis, glomerulosclerosis, and proteinuria. Concurrently with mesangial expansion, the fenestrations between podocyte foot processes also expand, decreasing the available surface area for filtration. Furthermore, monocyte chemoattractant protein-1 (MCP1) attracts activated macrophages/monocytes into the renal tubulointerstitium, leading to glomerular endothelial membrane leakage and renal injury. Polyol pathway, and phosphoinositide-3-kinase/protein kinase B (PI3K/Akt) signaling are implicated in the process of glomerular hypertrophy and podocyte injury. Under high glucose conditions, mesangial cells cumulate polyols, and produce an excess of matrix proteins. The accumulation of extracellular matrix proteins, specially collagen and fibronectin, has been attributed for thickening of glomerular membrane, and mesangial matrix expansion in DN. Upregulation of collagen expression in renal tissues promotes accumulation of mesangial matrix proteins, thereby inducing glomerulonecrosis. Moreover, dilation of the fenestrations tend to make the filtration system leaky. Thus, larger molecules e.g. proteins filter out of the blood leading to one of the prime markers of DN, i.e. albuminuria (24). CD80, an immune-related molecule, is directly involved in focal segmental glomerulo-sclerosis and proteinuria via integrin downregulation.

As a combined result of all the factors, ischemia, cell death, and atrophy of the vasculature supporting glomerulus and tubules come into effect over time. This, inevitably results in decline of kidney’s ability to filter blood, gradually leading to renal failure. Interestingly, expression of extracellular matrix proteins in kidney is negatively regulated by store-operated Ca^2+^ channels in mesangial cells, which may act as an endogenous renoprotective strategy in diabetics (25). Importantly, all the contributing factors to DN are directly associated with underlying hyperglycemia. Therefore, progress of DN can potentially be slowed down and/or prevented by controlling DM.

## 3 Rationale to use nanotheranostics in DN

The use of nanotechnology for the diagnosis and treatment of human diseases has increased dramatically in the recent decades owing chiefly to their capacity to function as theranostic tools that can carry a therapeutic load, and/or improve image contrast in diagnostics (26). Nanoparticles are utilized to deliver both small-molecules and big macromolecular (proteins, and nucleic acids) medicaments, as well as to diagnosis and detect disease progression. Nanoparticles have gained importance as novel designed nanomedicine due to their outstanding biocompatibility, tiny size to cross the cell barrier, wide surface area to carry effective drugs with high loading, and possibility of selective targeting of the drug to the affected area (27). Emerging evidence revealed that nanoformulations are able to reduce the necessary dose, increase permeability, and alter solubility to achieve maximal bioavailability and therapeutic efficacy (28, 29). Nanoparticles, which are preferable candidates for loading and delivering poorly soluble drugs, have been found to improve bioavailability and maintain long-term blood circulation to facilitate drug(s)’ sustained release. Because of the kidneys’ natural ability to choose nanoparticles within a stipulated size range, limiting the size of nanoparticles within a specific range is a design criterion for creating nanoparticle-based therapeutics targeting renal diseases. Particularly, nanoparticles with sizes smaller than 10 nm pass past the glomerular filtration barrier, whereas those larger than 100 nm seldom disseminate into the kidney because they are primarily retained by the liver and spleen. On the contrary, during DN, the kidney’s mesangium can sequester nanoparticles between 30 and 80 nm in size, reducing liver retention and hepatotoxicity (30).

Many potential therapeutic candidates exhibit poor therapeutic index and pose difficulties for formulation scientists. In an effort to address these issues, nanosized formulations are seen as superior and safer alternatives to conventional formulations. Nanotechnology-based techniques not only increase the surface area of pharmaceuticals, but also somewhat change the physiochemical characteristics of the active components (31). The overall benefit is shown in improved efficacy, improved dose management, and lower dose. Nanoparticles are well suited to improve absorption for drugs that undergo extensive first-pass metabolism on oral administration. The physiochemical features of the nanoparticles, their distribution and binding characteristics, plasma concentration, urine pH, biological variables, diseased state, and blood flow to the kidneys all influence clearance. Overall pharmacokinetic properties depend on particle size, surface charge, shape, surface morphology, surface engineering etc. Pharmacokinetic studies that take into account the materials of nanoparticles as well as the loaded drugs are the need of the hour to better predict the future of nanomedicine.

**Figure 2:**
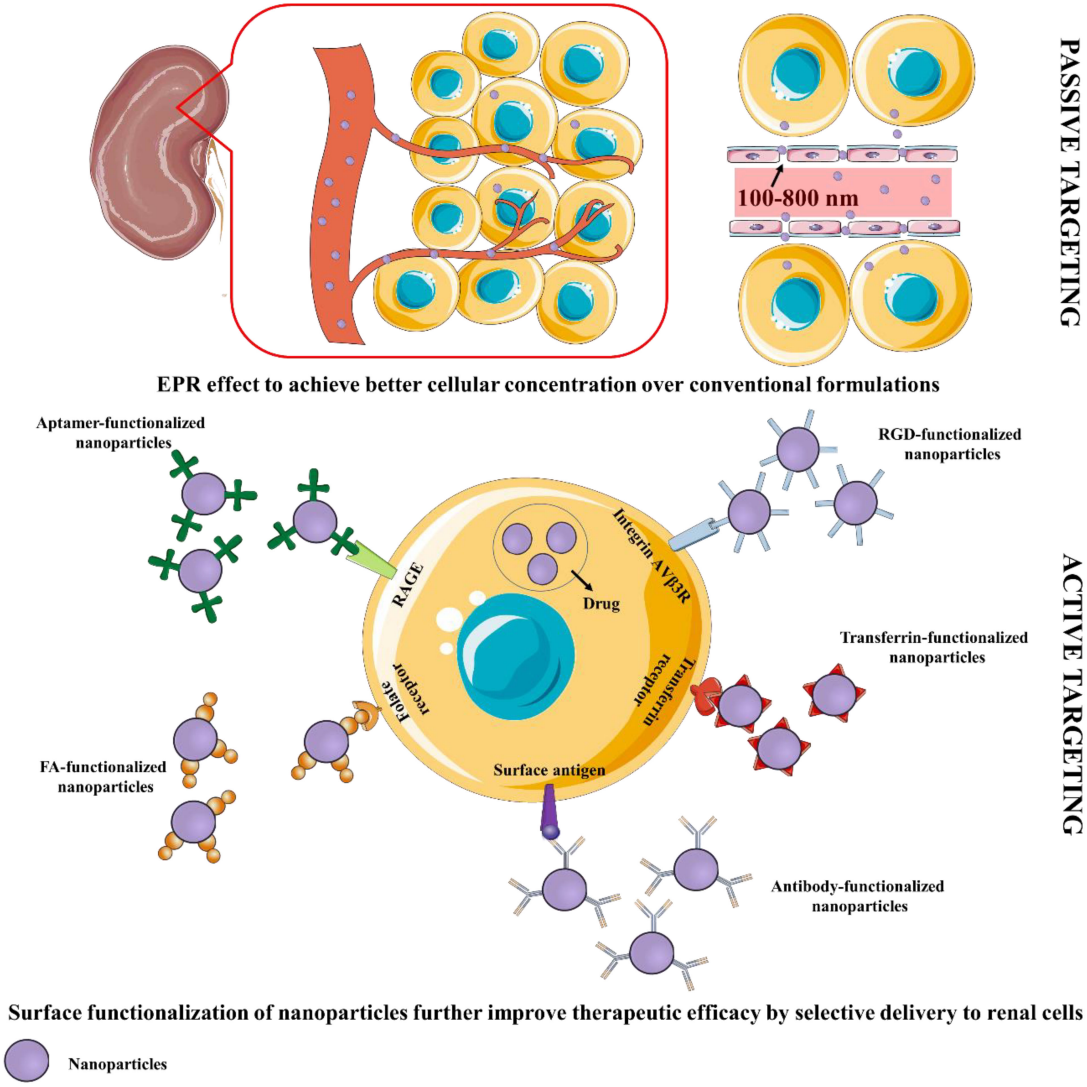
Schematic representation of passive and active targeting of renal cells by nanoformulations. EPR, Enhanced permeability and retention; FA, Folic acid; RGD, Arginylglycylaspartic acid.

Many therapeutic moieties exhibit promise in treating DN-related complications. However, poor solubility profile, poor bioavailability, low permeation, and high therapeutic dose apprehend potential clinical utilization (16, 29). Different nano-scale formulations, present with efficient targeting, and reduce dose-related toxic effects (29, 30). Nano-scale delivery systems offer numerous benefits to overcome the shortcomings of potential therapeutic agents (32). Formulations can be designed for reaching the target site at desirable concentrations. Passive targeting can be achieved by designing formulations to reach the target region mainly by utilizing the enhanced permeability and retention (EPR) effect (**Figure 2**). Formulations must remain in the circulation for a long time permitting their transmission to the target receptor(s). Certain aspects like pH, temperature, molecular size, microenvironment etc. can aid to achieve the same. External stimuli viz. ultrasound, hyperthermia, light, electric or magnetic fields may also contribute to regulate and/or trigger activity of nanosystems. Regarding renal delivery, due to increased vascular permeability and localized inflammation in the nephritic state, nanoparticles are more likely to house in the glomerulus (33). Active targeting is based on the affinity of ligand to specific receptor. In this approach, delivery of a certain quantity of either a therapeutic agent or a diagnostic agent or both, to target cells is based mainly on ligand-receptor interactions (34). A number of targeting ligands such as antibodies and non-antibodies (transferrin, RGD, folic acid, etc.) have been utilized to endorse selective delivery of the nanoformulations to particular regions (**Figure 2**). The presence of several targeting windows within the sick nephron, such as size cutoff, presence of charge-bearing components, and availability of particular receptors, can be used to develop functional nanoparticles for targeted therapeutic and diagnostic reasons. Current DN treatment options focus more on blood pressure, glycemia, and cholesterol management compared to molecular progression mechanisms of DN. Given the recognized difficulties, recent research suggests that nanoparticle-based platforms may provide potential futuristic ways to combat debilitating disorders such as DN. Several nano-systems have been developed that can be tagged/conjugated with certain moieties for improved targetability and/or traceability.

## 4 Formulation aspects for renal delivery

**Figure 3:**
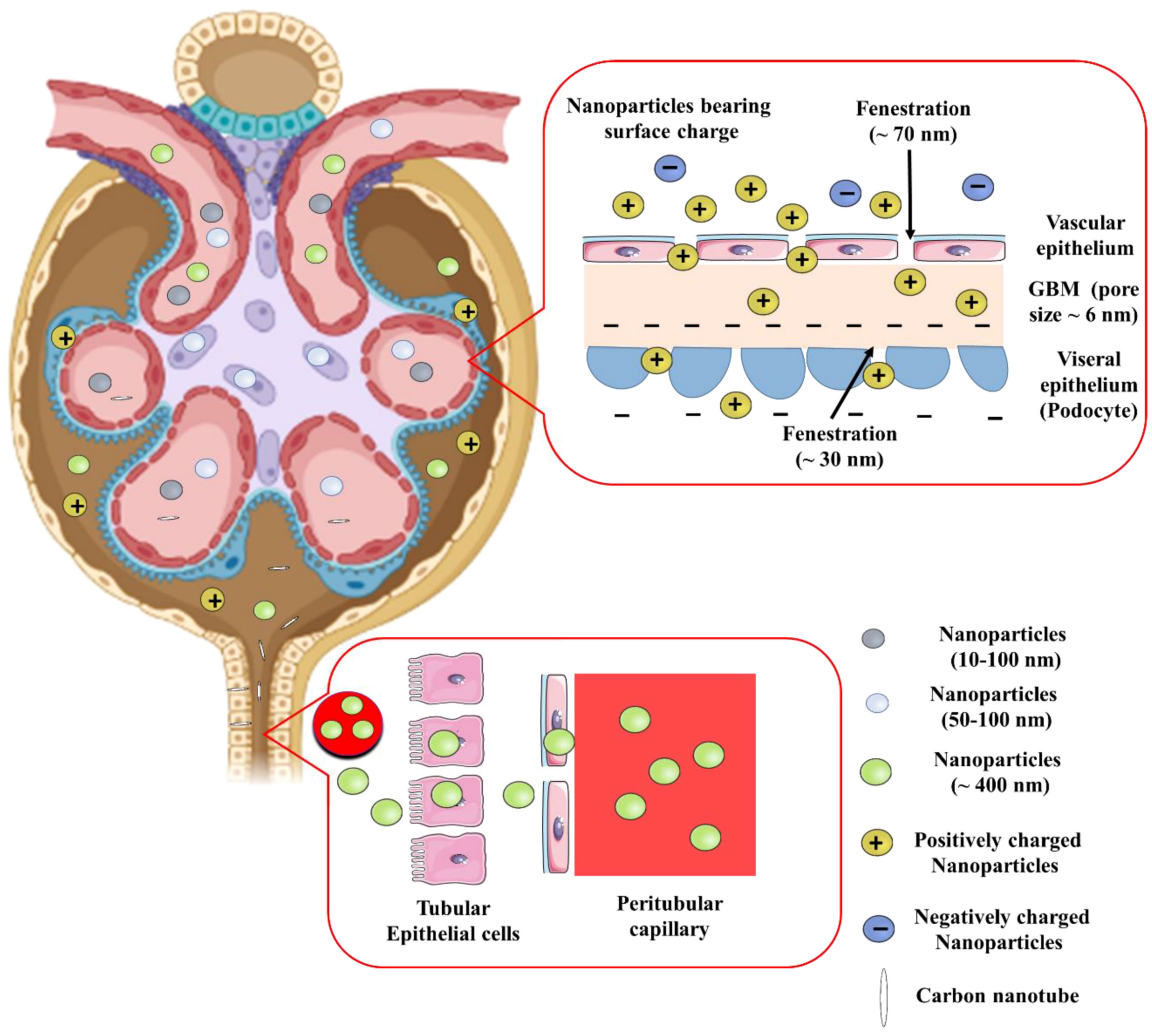
Effect of size, charge, and shape of nanoparticles on glomerular filtration barrier for renal retention. GBM, Glomerular basement membrane.

The glomerular basement membrane, podocytes, mesangial cells, and proximal tubules are the key sections of the kidney implicated in nephropathy. Many parameters, including size, charge, protein conjugation, and receptors, protect the formulations’ desirable site of action (**Figure 3**). Make, size, and interaction of the nanoparticles impact the biodistribution significantly.

### 4.1 Size

Due to the kidney’s natural ability to act as a blood filter, smaller diameter nanoparticles (<10 nm) are quickly eliminated from the kidney. The glomerular endothelium, with pores of about 70 nm, is the initial part of the three layer filtration system in glomerular area of nephron. The glomerular basement membrane comprises of an interwoven meshwork that can filter tiny molecules based on their charge and size. Along with this membrane, podocytes with filtration slits of approximately 30 nm also contribute. Water and minute plasma molecules can pass through the glomerulus, which filters blood depending on size and charge. The blood still contains higher molecular weight compounds and anionic charged components e.g., albumin (Albuminuria is brought on by DN-related disruption of this barrier). Therefore, structural characteristics of nanoparticles are crucial for their ability to reach renal cells. The nanoparticles with the highest plasma half-life have a particle size of about 100 nm. Smaller particles are more likely to pass through the kidneys. Larger nanoparticles (> 100 nm) are unable to reach the renal mesangium because of the size restriction imposed by the fenestrations, whereas nanoparticles with a diameter of about 75 ± 25 nm were specifically aimed towards the renal mesangium. On the contrary, proximal tubules can selectively be targeted with nanoparticles as large as 400 nm (35).

### 4.2 Charge

Because of their greater ability to attach to proteins and interact with the mononuclear phagocytic system, positively charged nanoparticles are rapidly eliminated from the kidney. Macrophages preferentially absorb negatively charged nanoparticles compared to neutrally charged nanoparticles. Nanoparticles with <15 mV charges are less likely to be taken up by macrophages, and display better circulation in blood (36). Cationic nanoparticles are easily taken in by the cellular membrane, and are capable of endosomal escape. As a result, negatively charged glomerular basement membrane easily absorbs positively charged nanoparticles to neutrally charged nanoparticles due to its anionic composition. On the contrary, ultrasmall (<5nm) nanoparticles with negative surface charge had been discovered to enter the glomerular capillaries, where they were able to bypass the glomerular endothelium and slowly accumulate in mesangial cells for 30 days (37). This, further supports the the primary role of charge in determining renal uptake of nanoparticles smaller than 5.5 nm.

### 4.3 Shape

Besides the key attributes like particle size and surface charge, the shape of nanoparticles influences drug targeting, even if the particle size is not perfect given the points discussed earlier. This is especially true for carbon nanotubes due to their higher aspect ratio (38). When the spatial orientation of the carbon nanotubes is perpendicular to the basement membrane, their lower diameter and higher aspect ratio enable them to pass through the glomerulus (39). Similar-sized nanoparticles usually have varied densities, which affects the speed and distribution through blood circulation. While low density nanoparticles circulate more quickly in the bloodstream, leading to quicker kidney clearance, shorter blood retention, and lower targeting; most of the dense nanoparticles do tend to have higher buoyancy forces and do not stay in the centre of the bloodstream where the speed is more, thus approaching the blood vessel walls more quickly.

### 4.4 Materials of construction

Nanoparticles composed of phospholipids, biologically derived lipids, natural polymers and strategically designed biodegradable polymers, and dendrimers are regarded as biocompatible and devoid of unwanted effects. However, polymeric nanoparticles are usually more prone to hepatic metabolism. Polymeric nanoparticles may be effective in overcoming the renal filtration threshold due to their low molecular weight, which allows them to be filtered through the kidneys and retained in the kidneys via post-glomerular processes. The tendency of biological nanoparticles to be more kidney-specific leads to an improvement in efficacy; examples include those that mimic the influenza A virus’s sequence recognition technique (40). Poly(vinylpyrrolidone-co-dimethyl maleic acid) has been demonstrated to have a high accumulation potential in the kidney and can be conjugated with superoxide dismutase for the treatment of renal diseases (41). Poly-l-glutamic acid is a renal polymeric drug carrier that is selective for renal protective drugs and has advantages such as high drug loading, non-immunogenicity, biodegradability, and biocompatibility (42). Many cases, nanoparticles are coated with polymers like polyethylene glycol (PEG), polyethylene oxide, dextran, polysorbates, and starch and small molecules like citrate to elevate biodistribution. Due to their decreased propensity for protein adsorption, PEGs in the mass range of 10 kDa are thought to make for superior coating materials than other PEGs (31, 43). Apolipoprotein E binds to polysorbate 80 to facilitate the transport of polysorbate-coated nanocarriers. Polysorbates also act as P-gp inhibitors to improve permeability of nanoparticles (44). Biodegradable nanoparticles e.g. poly(lactic-co-glycolic acid) (PLGA) nanoparticles are relatively easily metabolized, and the degradation products can be used in biological cycles like the Krebs cycle. Surface modification with poloxamers and/or citrates can enhance the circulation time of nanoparticles.

### 4.5 Targeting

Various techniques have been utilized for targeting renal cells based on their location in the kidney. Site-specific delivery of customized nanoparticles improves therapeutic impact, and decreased harmful side effects. In many cases, *in vivo* administration of nanoparticles in tumors has been the subject of several investigations, which led to few accidental findings of kidney-selective targeting and accumulation (38, 45). In addition, numerous biological ligands can be utilized to target precise areas, and improve specificity and efficiency, thus avoiding systemic accumulation and potential toxicities. The ligand, on the other hand, may be accountable for an unexpected deviation from the normal physiological pathway required for renal uptake, affecting the clearance and targeting. Indeed, the selection of the ligand is influenced by factors such as size, shape, hydrophilicity, immunogenicity, and stability, in addition to specificity. Nanoparticles can be modified to increase its effectiveness as well as target-specific distribution by polyethylene glycol (PEG)ylation, peptide conjugation, alterations in surface chemistry, and ligand binding. Angiotensin II receptors, megalin receptors, collagen IV, and v3 integrin receptors have demonstrated promise as possible targets for target-specific drug delivery to treat DN (46). Futuristic theranostics using nanoparticles have shown promise for treating a wide range of illnesses and ailments.

## 5 Nanoformulations in management of DN

**Figure 4:**
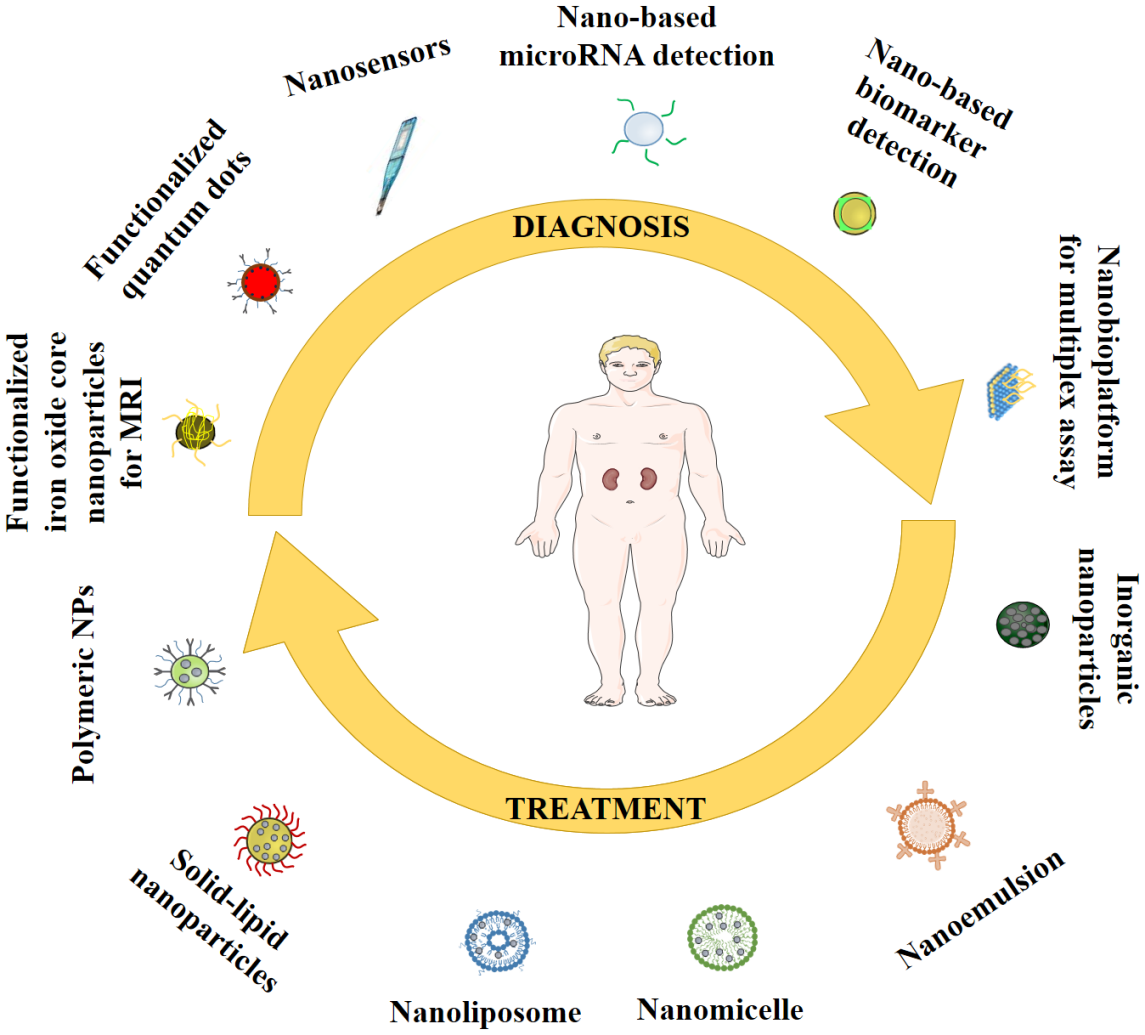
Different roles of nanoparticles in the management of DN.

Advancements in nanotechnology have enhanced the scope for early diagnosis of a disease including DN. Many therapeutic molecules exhibit promise in treating DN complications. However, clinical relevance is constrained by their poor solubility profile, poor bioavailability, poor permeability, high therapeutic dose, and related toxicity. Numerous studies have demonstrated that nanoparticles, with their enhanced pharmacokinetic properties and targeting capabilities, offer a high potential for treating DN (47, 48). Different potential roles of nanoparticles in management of DN are schematically represented in **Figure 4**.

### 5.1 Diagnosis

Since many diabetic complications do not display clinical symptoms immediately, systemic screening based diagnosis at early stages is the need of the hour. The monitoring of kidney status using a number of non-invasive imaging modalities, including as computed tomography (CT), positron emission tomography (PET), and magnetic resonance imaging (MRI), is now being used, however, applications of nanotechnology-hyphenated non-invasive imaging modalities have been found to be proficient enough for accurate diagnosis of DN.

A variety of magnetic nanoparticle-based probes have been created as contrast agents for imaging. Due to their exceptional magnetic and biodegradable qualities, iron oxide nanoparticles are amongst the most researched nanoparticles for enhancing clinical imaging. However, because they are biocompatible, superparamagnetic iron oxide nanoparticles (SPIONs) are more compelling. These nanoparticles can be targeted with magnetism, tracked using MRI, and even be used as magnetic triggers for drug release thanks to their superparamagnetic capabilities (49, 50). Emphasizing the fact that early diagnosis can lead to higher chances of therapeutic success, utilizing the role of low-density lipoprotein receptor 1 (LOX-1) as a potential biomarker, anti-LOX-1 superparamagnetic iron oxide PEG-coated nanoparticles have been designed to identify inflammatory renal lesions during early stages of DN (51). It has been shown that superparamagnetic iron oxide PEG-coated nanoparticles demonstrates good cellular internalization and durable MRI tracking abilities. The study by Luo and colleagues (45) supplies important informations with regard to detection, Characterization, and monitoring of early DN, utilizing the interaction between LOX-1-enriched inflammatory renal lesions and anti-LOX-1 targeted nanoparticles. However, more research is necessary on the toxicity and safety of LOX-1 targeted superparamagnetic iron oxide PEG-coated nanoparticles. Gold nanoparticles (AuNPs) are a promising choice for improving CT imaging because of their capacity to increase X-ray attenuation. AuNPs were found to greatly increase the contrast to noise ratio of CT in an imaging phantom at both low (40–60 kVp) and high (100–140 kVp) tube potentials compared to the clinically utilized contrast agent iodine (52, 53).

Quantam dots (QDs) also display futuristic prospects in diagnosis purpose. QDs can be endorsed as multimodality contrast agents used in CT, PET, MRI, infrared fluorescence, and photoacoustic imaging applications (54). In a study, occurrence of aldose reductase (AR) and toll-like receptor 4 (TLR4) in cells and renal tissues of diabetic rats was identified by a QD-based immunofluorescence technique and compared with the conventional immunohistochemistry method (55). For this purpose, CdSe/CdS/ZnS QDs were prepared and conjugated with rabbit polyclonal TLR4 antibody and mouse monoclonal AR antibody. Study results explained that both the AR and the TLR4 proteins were upregulated in the renal tissues of diabetic rats. The relationship between AR and TLR4 in the pathogenesis of DN was revealed by the developed dual-colour QD-based immunofluorescence labelled technique to study the expressions of AR and TLR4 in the renal tissues simultaneously. Compared to the conventional immunohistochemistry that require 7-9 h to provide 100% detection rate, the QD-conjugated with primary antibody and conventional immunohistochemistry shows maximum detection rate in minimal time of 3.5 h making it more convenient regarding accuracy (55). Summarily, this QD-based multiplexed imaging method offers new insights into the mechanistic investigation of biological component correlations, as well as possible applications in disease diagnosis and treatment. In another experiment, mono-dispersed and size controllable ^89^Sr-doped CdSe QDs were prepared and coated with polyamidoamine to improve the accuracy and stability of quantitative analysis of renal injury of DN (56). The biocompatible QDs, besides facilitating urinalysis-based detection of renal injury markers through both fluorescent and radio-analytical methods, did not elicit any side effect. Clearly, the QD-based imaging methods offer a new vision into the mechanistic study of the correlation among biological factors with DN.

Due to its stability and ease of detection in human urine, microRNA (miR) can also be utilized to identify DN (57). Urinary exosomal miR is identified in early-stage DN. MiR-192, miR-21, miR-770, and miR-126 are also employed as biomarkers (58). Exosomal urine miRs shielded from RNase activity have steadily developed into a reliable novel biomarker for the detection of chronic kidney disease in its early stages, a field in which metal nanoparticle-based diagnosis is becoming more and more pronounced. A simple colorimetric assay based upon AuNPs has already been designed by Nossier and colleagues (59). The method relies on quantification of certain urinary miRs viz. MiR-210 and miR-34a to detect DN. In another study, a simple, cost-effective, portable method has been developed to determine DN via silver nanoparticles (AgNPs)-based colorimetric determination of creatinine (60). In a recent development, AuNP-based 3D sensors have proved reliable to detect thiamine in urine which can be correlated with onset and progress of DN (61). These methods present with advantages like simplicity, high reliability, and cost-effectiveness. Even in few cases, portability emerges as an added advantage along with non-invasiveness.

### 5.2 Treatment

Nanoformulations have drawn growing interest for therapeutic purposes due to their potential to cross biological barriers and boost the bioavailability of the drugs. With advancements in biochemistry and nanotechnology, antidiabetic medications can be disintegrated, implanted, encapsulated, or attached to nanoparticles using a nanoplatform for drug delivery. Additionally, active targeting with nanoparticles might also help with effective and site-specific delivery of therapeutic agents. **Table 1** enlists potentially useful nanocarriers for treatment of DN.

**Table 1:** Different nanoformulations in DN therapeutics.

S. No.	Nanocarriers	Key components/features of nanoformulations	Therapeutic agents	Outcomes	References
1	Liposomes	SAINT C18	Rapamycin	Improved podocyte targeting with rapamycin	(62, 63)
2	Liposomes	Ultrasound-targeted microbubble destruction	Co-enzyme Q10	Preservation of podocytes, inhibition of apoptosis	(64)
3	Liposomes	soybean phosphatidylcholine, sodium deoxycholate	Eprosartan mesylate	Renal protection via blocking angiotensin II receptors	(65)
4	Liposomes	Ultrasound targeted microbubble destruction	Basic fibroblast growth factor	Intrarenal delivery, inhibition of inflammation	(66)
5	Liposomes	polymeric core and lipidic shell modified with kidney targeted peptide	Rhein	Improved kidney targeted distribution, and bioavailability	(67)
6	Liposomes	Distearoyl phosphatidylcholine	Hirudin	Enhanced accumulation of hirudin in renal tissue to relieve renal injury	(30)
7	Liposomes	Glucose-ligand conjugation	Astaxanthin	Preferential renal distribution via overexpressed GLUT1 receptors on mesangial cells	(68)
8	Liposomes	PEGylation	Quercetin	Renal protection	(69)
9	Nanoliposomes	–	Silymarin	Attenuation of DN-associated renal injury	(70)
10	Nanoliposomes	soybean phosphatidylcholine	Calycosin	Regulation of mitochondrial respiratory activities	(45)
11	SLNs	Compritol	Myricitrin	Improved bioavailability of myricitrin	(71, 72)
12	NLCs	Glyceryl monostearate and decanoyl/octanoyl-glycerides	Ergosterol	Improved oral bioavailability against DN complications	(73)
13	Niosomes	Cholesterol	Gymnemic acid	Inhibition of AGEs and oxidative stress	(74)
14	Niosomes	Cholesterol	Rubiadin	Oral admi istration, improved biomarker status	(75)
15	Polymeric nanoparticles	Chitosan	siRNA	Kidney-specific delivery utilizing megalin receptors	(76)
16	Polymeric nanoparticles	PLA-P85-PLA	Insulin	Protection of insulin from enzymatic breakdown, improved permeability	(77)
17	Polymeric nanoparticles	Cyclodextrin	siRNA	Greater uptake of mannose-targeted nanoparticles in mesangium	(78)
18	Polymeric nanoparticles	PEG-*b*-(PELG-*g*-PZLL)	Quercetin	Downregulation of ICAM1 on renal epithelium	(79)
19	Lysozyme-conjugate PEI nanoplexes	PEI	Gentamycin	Megalin-mediated uptake in proximal cells	(80)
20	Polymeric nanoparticles	Polyethyleneglycol-co-polycaprolactone-co-polyethylenimine	Rhein	Improved solubility, and renal distribution of rhein	(81)
21	Polymeric nanoparticles	PLGA	Phe-Tyr dipeptide	Improved bioavailability of peptide	(82)
22	Polymeric nanoparticles	Chitosan	Insulin	Oral delivery reduced fasting blood glucose level	(83)
23	Polymeric nanoparticles	PLGA	Crocetin	Anti-inflammatory, antifibrotic activities	(84)
24	Polymeric nanoparticles	Chitosan	Polydatin	Stronger hypoglycemic response and amelioration of DN compared to native polydatin	(85)
25	Polymeric nanoparticles	PLA	*Tinospora cordifolia* stem extract	Reduced expression of proinflammatory cytokines	(86)
26	Polymeric nanoparticles	Chitosan	Berberine	Improved permeability of berberine	(87)
27	Polymeric nanoparticles	p(AAPBA-b-HPA)	Insulin	Glucose and pH-sensitive delivery of insulin	(88)
28	Polymeric nanoplex	Dendrimer-templated polymer, albumin	Si RNA	Enhanced serum stability, avoidance of degradation by RNase, cytosolic delivery of siRNA following endosomal escape	(48)
29	Lipid-polymer hybrid nanoparticles	Chitosan, tripolyphosphate	All-trans retinoic acid	Improved solubility profile of retinoic acid	(1)
30	Metallic nanoparticles	AuNPs	Pomegranate peel extract	Downregulation of ROS production	(89)
31	Metallic nanoparticles	AgNPs	*Momordica charantia* leaf extract	Normalization of KIM1 levels	(90)
32	Metallic nanoparticles	AgNPs	*Momordica charantia* leaf extract	Reversal of DN-mediated alterations of gene expressions	(91)
33	Nanoparticles	*In situ* AuNPs onto chitosan functionalized PLGA nanoparticles	Insulin	Long-term insulin release upon oral delivery	(92)
34	Mesoporous silica nanoparticles	Silica, cerium oxide	Metformin	Long-term cyclic scavenging of free radicals	(93)
35	Biological nanoparticles	Virus	Cinaciguat	Active accumulation of cinaciguat at mesangial sites	(40)
36	Cationic nanoparticles	Cyclodextrin, adamantine-PEG	siRNA	Electrostatic interaction with negatively charged glomerular 4444rrrr4basement membrane	(36)
37	Immunoliposomes	Hydrogenated soybean phosphatidylcholine	Mycophenolate mofetil	Size-dependent retention in mesangium resulting in decreased cell expansion	(94)
38	Nab-paclitaxel	Albumin	Paclitaxel	Renoprotective effects	(95)
39	Nanoparticles	PF-A299-585	Triplotide	Renal targeting	(96)
40	Nanoparticles	Albumin	Methylprednisolone	Neonatal Fc receptor-targeted delivery improved cellular uptake in podocytes	(97)
41	Nanoparticles	Melanin@Glc-NCM	Melanin	Improved bioavailability of melanin, photo-thermal and glucose-sensitive delivery to mesangial cells	(98)
42	QDs	Semiconductor	RGDfC motif	Enhanced binding to αvβ3 receptors on podocytes	(99)
43	Metallic nanoparticles	ZnO nanoparticles	–	Activation of autophagy, elevated antoxidant and anti-inflammatory effects	(100)
44	Metallic nanoparticles	AuNPs	AuNPs combined with dapagliflozin	Renoprotective effect by targeting miR-21 and miR-192	(101)
45	Polymeric nanoparticles	PGA-coated polymeric nanoparticles	Rhein	Improved renal drug distribution and cellular uptake	(102)
46	Metallic nanoparticles	SeNPs	SeNPs combined with bee venom	Improvements in biochemical, histological, and molecular parameters	(103)
47	Nanoparticles	p-Coumaric acid nanoparticles	Chitosan	Improved nephroprotection	(104)
48	Metallic nanoparticles	AuNPs	–	Attenuation of high glucose-induced cytotoxicity on renal tubular epithelial cells	(105)

#### 5.2.1 Lipid-based nanoparticles

Lipid-based nanoparticles are regarded as highly promising drug carriers due to their benefits of biodegradability, biocompatibility, higher kidney retention, and minimal immunogenicity. Liposomes, in particular present themselves with certain advantages like improving membrane permeability, sustained release properties, and site-specific delivery via surface functionalization. Among lipid-based formulations, liposomes offer the highest biocompatibility and biodegradability (28). Liposome systems, however, face significant challenges in terms of long-term stability. PEG chains can be covalently linked to increase liposome stability and *in vivo* circulation time (106). Solid lipid nanoparticles (SLNs) have been created as an alternative to liposomes in order to address their stability issues. Surfactants are essential parts of SLNs at the binding interface and lower the interfacial energy between the lipid and aqueous phases. The somewhat imperfect crystalline form of Nanostructured lipid carriers (NLCs), on the other hand allows for more drug loading, prevents drug outflow owing to lesser water content.

Improved renal hemodynamics during early stages of DN has been observed with co-enzyme Q10 containing liposomes in combination with ultrasound targeted microbubbles destruction (64). Upregulation of Bcl-2, and downregulation of Bax and caspase 3 indicated low to no cytotoxicity, and preservation of podocytes. Another group of researchers claimed improved delivery of co-enzyme Q10 to kidneys by formulating into liposomes, also utilizing ultrasound targeted microbubbles destruction for site-specific delivery (107). With an entrapment effectiveness of 86.15%, co-enzyme Q10 was efficiently encapsulated within the liposomes exhibiting hydrodynamic diameter of about 180 nm and a negative surface charge. Coupling of liposomes with ultrasound-mediated microbubble destruction clearly improved kidney-specific distribution. These studies establish co-enzyme Q10-liposomes in combination with ultrasound microbubbles as a potential strategy to prevent and/or reverse the progress of early DN. Similar improvement was also reported with intra-renal delivery of basic fibroblast growth factor-loaded liposomes utilizing ultrasound targeted microbubbles destruction (66). Inflammation-mediated cellular apoptosis of renal tubular cells was significantly minimized too, on treatment with the formulated liposomes. Clearly, developed formulation played crucial roles to overcome the earlier known shortcomings of basic fibroblast growth factor i.e. short half-life, low stability and poor penetration.

Oral delivery of eprosartan mesylate-incorporated bilosomes exhibited better therapeutic efficacy to protect murine diabetic kidneys evidenced by the improved reduction of serum creatinine, total albumin, urea, lactate dehydrogenase, and malondialdehyde levels over eprosartan mesylate suspension, which has been achieved by the superior blockade of angiotensin II receptor by this novel nanoformulation as compared to conventional suspension (65). Bearing negative surface charge, the bilosomes were favourably taken up by Payer’s patches. In the future, the produced eprosartan mesylate loaded nano-bilosomes could serve as a feasible oral formulation for DN and may give prospective benefits in situations of hypertension and renal illness. Liposomal hirudin has been found to be more effective than native hirudin in DN, acting by inhibiting the expression of vascular endothelial growth factor (VEGF) and TGF-β1 in the diabetic kidneys (30). In another interesting experiment, targeted delivery of astaxanthin has been achieved by using glucose ligand-conjugated astaxanthin-loaded liposomes that are transported by overexpressed glucose transporter 1 (GLUT1) transporter on the cell membrane of glomerular mesangial cells (68). Glucose-PEG600-DSPE ligand modified liposomes could specifically transport on the membrane of glomerular mesangial cells by overexpressed GLUT1, and achieved excellent kidney-targeted drug delivery. PEGylated liposome containing quercetin exhibited renal protective effects in DN, by attenuating oxidative stress, reducing AGE expression, and delaying the progression of DN (69). Lipid nanovesicles have been prepared with l-α-phosphatidylcholine, 1,2-distearoyl-sn-glycero-3-phosphoethanolamine-N-[biotinyl(polyethylene glycol)-2000] and cholesterol that selectively target Tm cells. However, the kidney biopsies from the patients with lupus nephritis, patients with DN, and healthy controls demonstrated that the kidneys of patients with lupus nephritis are highly infiltrated with active CD8+ Tm cells with increased Kv1.3 expression (108). Higher expression of GLUT occurs in kidneys of diabetic population, which has been targeted by developing liposomes modified by mannose-PEG-distylacylphosphatidyl ethanolamine. Mannose aids in improving the therapeutic efficacy by augmenting the aqueous solubility of a drug (109). Kidney-targeted delivery of astaxanthin improved the bioavailability of astaxanthin and led to amelioration of renal pathological conditions by potentiating antioxidant capacity of the drug. Incorporation of glutathione into liposomal formulation improved therapeutic efficacy of glutathione by preventing oxidation of glutathione, thus enhanced antioxidant activity to suppress oxidative stress to nephrons involving polyol pathway (110). Silymarin nanoliposomes exhibited *in vivo* therapeutic potential in attenuating DN evidenced by decreasing renal fibrosis, inflammation and oxidative stress in DN rats mediated through TGF-β/Smad and JAK2/STAT3/suppressor of cytokine signaling (SOCS)1 co-suppression, leading to improved survival rate (70). This study demonstrated that the gap between *in vitro* and *in vivo* models arising due to poor pharmacokinetic attributes of a drug could be overturned through employing nanocarrier-mediated mediated advanced drug delivery tool. Incorporation of calycosin within liposomes improves the solubility and bioavailability (~2.3 times) than that of the free calycosin and thus enhanced therapeutic efficacy against DN (45).

SAINT-o-some, a modified liposome have already demonstrated improved serum stability and drug release characteristics. In addition, functionalization of SAINT-o-somes with particular antibodies like anti- vascular cell adhesion molecule 1 (VCAM-1) could improve therapeutic efficacy of SAINT-o-somes via active targeting to the podocytes (62). SAINT-O-somes generated tiny particles (106 nm) with 71% siRNA encapsulation effectiveness. SAINT-O-somes were serum stable at 37°C, protected siRNA from destruction by serum RNases, and had pharmacokinetics equivalent to standard long circulating liposomes following intravenous administration. These anti-VCAM-1 and anti-E-selectin SAINT-O-Somes are thus a unique drug delivery system capable of delivering siRNA into inflammed primary endothelium cells and possessing physicochemical properties to meet the needs of potential clinical application. VCAM-1 has been regarded to be expressed in association with tumor necrosis factor α (TNF-α) activation. Thus, cellular absorption of targeted SAINT-o-somes would to be greater than that of non-targeted SAINT-o-somes during TNF-α overexpression. In this aspect, rapamycin could be used for active targeting TNF-α-overexpressed podocytes (63). Clearly, anti-VCAM-1-rapamycin-SAINT-O-Somes reduced AB8/13 cell migration more effectively than free rapamycin and non-targeted rapamycin.-SAINT-O-Somes demonstrating the viability of VCAM-1 focused drug delivery to podocytes. Myricitrin is well-known to decrease oxidative stress-mediated cytotoxicity; however, it has poor bioavailability issues to meet with desired therapeutic effect *in vivo*. In a study, mycitrin-loaded SLNs improved DN altercations more prominently by lowering oxidative stress and raising antioxidant enzyme levels, compared to native myricitrin, which clearly demonstrated that the mycitrin-loaded SLNs could abrogate the bioavailability issue with mycitrin (71). The DN model used in this study is not well established, which sparks ambiguity over the practical impact of the outcomes (72). However, that does not seem to impact the improved efficacy of myricitrin on being encapsulated within SLNs. Formulation into NLCs also enhanced the therapeutic efficacy of ergosterol in DN by improving solubility profile as well as oral bioavailability of the drug (73). The spherical, negative surface charge-bearing formulation encapsulated ergosterol to a very high extent, and exhibited promise to improve therapeutic efficacy of the same.

#### 5.2.2 Niosomes

Niosomes are liposome-like vesicles composed of single chain surfactant molecules along with cholesterol. Niosomes are physically and chemically more stable with additional cost-effectiveness, and ease of scale up (111, 112). Gymnemic acid niosomes have been proven to be a successful drug delivery tool to attenuate DN in rats by inhibiting oxidative stress, inflammation, and AGE formation (74). In another preclinical study, oral administration of rubiadin-loaded niosomes exhibited therapeutic promise against DN in rats (75).

#### 5.2.3 Polymeric nanoparticles

Polymeric nanoparticles have piqued the interest of many researchers in the realm of medical biology owing to their high efficiency, ease of synthesis, long circulation time, biocompatibility, low toxicity, ability to absorb/encapsulate, and carry other molecules, and possibility of active targeting via surface modification (113, 114). Polymeric nanoparticles can deliver a wide range of drug(s) that offer high surface to volume ratio and have enormous opportunity to ameliorate DN by virtue of their versatile physicochemical properties. A wide array of polymers are promising choices because of their adaptability and flexibility in order to satisfy the needs of nanotechnology-based drug delivery. Additionally, they can be designed with a variety of desirable properties, which enhances their appeal, as does their capacity to tolerate physiological stress and their high biological stability. Polymeric nanoparticles can also be altered by ligand attachment and/or functionalization with surface modification for site-specific drug delivery.

Insulin-loaded PLA-P85-PLA vesicles have been proven to be effective in maintaining blood glucose homeostasis for a longer period of time (77). Insulin is protected from enzymatic breakdown in the gastrointestinal tract by PLA-P85-PLA vesicles. Moreover, P85 displays significant cell membrane permeability in the intestines due to its amphiphilic characteristics. A study showed quercetin-loaded polymeric nanoparticles to successfully ameliorate DN by downregulating intercellular adhesion molecular-1 (ICAM1) on endothelium of kidney, and reducing the CD11b+ myeloid cells accumulation (79). *Tinospora cordifolia* stem extract-loaded poly-lactic acid (PLA) nanoparticles have been reported to ameliorate DN by downregulating expressions of proinflammatory cytokines (86). PLGA nanoparticles loaded with Phe-Tyr dipeptide improved the bioavailability of the peptide drug to open up the scope to treat DN along with hypertension and cardiovascular diseases (82). Emerging evidence showed that PLGA-based nanoparticles engineered with kidney injury molecule 1 (KIM1) could improve renal targeting (46). In an interesting development, PLGA nanoparticles with particle sizes that are on the larger side could be retained in the kidneys (115). The nanoparticles were PEGylated to improve circulation time, and resulted in enhanced accumulation in the kidney. Rhein-loaded polyethyleneglycol-co-polycaprolactone-co-polyethylenimine nanoparticles were found to be effective in DN management, and enhance the therapeutic efficacy via kidney-targeted distribution of rhein (81). By resolving the limitations of low aqueous solubility and low kidney distribution of rhein, the formulated nanoparticles not only reduced the fasting blood glucose, serum creatinine, blood urea nitrogen, and urinary protein levels, but also decreased the intensity of oxidative stress by inhibiting TGF-β1 and endorsing the dephosphorylation of Smad2/3 signaling pathways. Rhein-loaded polycaprolactone-polyethyleneimine nanoparticles improved renal medication distribution and cellular uptake via poly-γ-glutamic acid (PGA)-mediated receptor-ligand interaction with –glutamyltranspeptidase (102). The PGA coating not only assured the stability, continuous drug release, and biocompatibility of the formed nanoparticles, but it also increased renal cellular absorption by interacting with-glutamyltranspeptidase on the renal cells. The PGA coating prevented the formulation from being recognized by the reticuloendothelial system, which resulted in a longer circulation period. As a result, the PGA coating method opens up a new path for the use of nanomedicine in the treatment of renal disorders including DN. Similarly, crocetin-loaded PLGA nanoparticles exhibited better therapeutic efficacy in the management of DN compared to free crocetin evidenced by superior anti-inflammatory, and antifibrotic effects compared to native drug (84). Recently, novel glucose-responsive nanoparticles for insulin delivery has been developed with a copolymer made of acrylic acid-P-hydroxyphenethyl anisate and a newly synthesized macromolecular initiator p(AAPBA) by block copolymerization (88). The nanoparticles made of the copolymer p(AAPBA-b-HPA) exhibited high sensitivity for both pH and glucose, high biocompatibility, good insulin loading and sufficient stability under physiological conditions. Furthermore, these nanoparticles showed therapeutic promise in controlling hyperglycemia and restoring renal function by mitigating oxidative and inflammation-induced renal injury in DN mice (88).

Chitosan, a non-toxic mucoadhesive polymer with biocompatibility and easy modifiability, has been found to be a reliable, valid intestinal permeability enhancer promoting protein drug absorption. Especially, low molecular weight chitosan has displayed selective accumulation in kidneys aiding in renal-targeted drug delivery (116). Chitosan nanoparticles have opened up a new possibility of oral insulin delivery to overcome drawbacks like needle phobia, and peripheral hyperinsulinemia. Oral delivery of insulin-loaded trimethyl chitosan nanoparticles depicted better solubility profile compared to chitosan nanoparticles. Study results indicated comparable reduction in fasting blood glucose levels with oral delivery of insulin-loaded trimethyl chitosan nanoparticles and injection of insulin. Additionally, nanoparticles also exhibited decrease in serum TGF-β1 levels (83). These findings indicated trimethyl chitosan nanoparticles loaded with insulin to be somewhat more effective regarding therapeutic intervention than insulin injection. This can be linked to the fact that, chitosan enhances mucosal adhesion and increases absorption through an ionic contact between its cationic amine groups and the anionic groups on the surface of epithelial cells,. As a result, intestinal epithelial tight junctions intermittently open to allow for inter-epithelial insulin transfer. When treating DN, polydatin-loaded chitosan nanoparticles have been demonstrated to improve therapeutic efficacy evidenced by stronger hypoglycemic, antioxidant, and anti-inflammatory effects over free polydatin (85). To achieve good clearance from the system followed by effective drug delivery, biodegradable chitosan nanoparticles came up with attractive outcomes. The improved therapeutic efficacy has been achieved through improving absorption and prolonged-release properties of polymeric nanoparticles are all part of the mechanism of the renal protective effects against DN. Encapsulation into chitosan nanoparticles has been demonstrated to successfully respond to biopharmaceutical limitations of berberine as an antidiabetic agent (87). Chitosan, by facilitating paracellular transport via transient opening of intercellular tight junctions, could do away with the problem of high P-gp efflux associated with berberine in native form.

#### 5.2.4 Lipid-polymer hybrids

These hybrid nanostructures usually consist of a polymeric core covered by lipid layer. They are constructed from at least two distinct materials to overcome the limitations of single component, and/or to achieve multiple functionalities for single nano-architectures. Hybrid nanoparticles offer distinct advantages like prolonged circulation time, high stability, biocompatibility etc. whereby lipidic and polymeric materials complement each-other. In a contextual study, rhein-loaded liponanoparticles (consisting of polymeric core and lipidic shell modified with kidney targeted peptide) improved cellular internalization by renal tubular cells, endothelial cells, mesangial cells, and podocytes via non-lysosomal pathways (61) (30). Novel two-step nanoparticular cascade of size control and enhancement of renal cellular uptake could successfully overcome the delivery obstacles to renal regions. In a later study, oral delivery of all-trans retinoic acid encapsulated chitosan/tripolyphosphate lipid hybrid nanoparticles improved therapeutic efficacy of retinoic acid against DN, as well as improved its solubility profile by nanoencapsulation (1). In comparison with free drug and drug-loaded lipidic nanoformulation, the hybrid nanoparticles under discussion exhibited superiority in ameliorating DN evidenced by the significant reduction of urea, creatinine, TNF-α, granulocyte-macrophage colony-stimulating factor, VEGF and ICAM-1 levels with increase in LKB1 and AMPK levels.

#### 5.2.5 Metallic nanoparticles

During the past few decades, metal-based nanoparticles have gained increasing attention in the biomedicinal field owing mainly to ease of synthesis, and long-term stability answering the low mechanical properties of polymeric nanoparticles. To avoid complexation of metal ions in the body, modification with sodium citrate can be very useful (117). In diabetic kidneys, selenium nanoparticles (SeNPs) treatment increased levels of heat shock protein (HSP-70), longevity protein Sirtuin 1 (SIRT-1), and regulated the apoptotic proteins Bax and Bcl-2 (118). SeNPs successfully inhibited apoptosis in kidney cells and prevented the course of DN not only by reducing oxidative stress and increasing the levels of cytoprotective protein HSP-70 and longevity protein SIRT-1. Chitosan-stabilized SeNPs have evolved as a potential therapeutic tool to ameliorate DN by downregulating expression levels of TGF-β1 and aldose reductase (119). Though vascular and glomerular congestion was observed in renal tissues of treated groups, the risk-benefit ratio seemed favorable. Mucosal adherence and increased absorption, results due to interaction of the cationic amine groups of chitosan with the anionic groups at the epithelial cell membrane (120). In a more recent study, citric acid and ascorbic acid-protected SeNPs ameliorated DN during pregnancy, and improve histopathological features of the kidney (121). Given that, the formulated SeNPs accelerated the start of gestation and improved rate of successful pregnancy among diabetics, the formulation seems to be a potential futuristic asset on establishing safety profile. SeNPS, in a very interesting study complemented bee venom to protect from DN by improving biochemical, histological, and molecular parameters (103).

Pomegranate peel extract-stabilized AuNPs downregulated production of reactive oxygen species (ROS) via blockade of protein glycation, and dephosphorylation of MAPK/NF-κB/STAT3-mediated proinflammatory response, thus creating an economic option to treat DN (89). AuNPs have been observed to limit expansion of mesangial matrix, and ameliorate podocyte damage to maintain permeability and integrity of glomerular filtration barrier (117). In another study, citrate-stabilized AuNPs exerted therapeutic effects against DN *via* ameliorating oxidative stress (105). Similarly, chitosan/sodium lignosulfonate AuNPs exhibited protective effects against DN (122). Clearly, fabrication of AuNPs enhanced antioxidant response while at the same time maintaining glycemic homeostasis. AuNPs (2.5 mg/kg/day, intravenous) can substantially ameliorate DN, in combination with dapagliflozin (2 mg/kg/day) (101). The treatment regimen successfully alleviated renal fibrosis, minimized apoptosis, and activated autophagy. They seemed to mitigate DN by targeting miR-192 and miR-21 and their downstream pathways i.e. fibrosis, apoptosis, autophagy, and oxidative stress. In line with the findings, AuNPs of about 30 nm diameter attenuated high glucose-induced cytotoxicity on renal tubular epithelial cells by downregulating free radicals, AGEs, and apoptotic marker proteins (105). However, the cytoprotective effects minimized in presence of SIRT-3 inhibitor.

In a study, *Momordica charantia* leaf extract-loaded AgNPs significantly elevated the level of HMG-CoA reductase, PPARα and PPARγ in DN subjects (90). Nanoparticles at a dose of 50 mg/kg for 12 days significantly upregulated PPARs compared to diabetic controls. The AgNPs also normalized level of KIM1. In another study, oral administration of *Momordica charantia* leaf extract-loaded AgNPs reversed DN-mediated upregulation of PI3K, Akt, TGF-β, JAK2, STAT3 and glucokinase genes, and consequent downregulation of phosphatase and tensin homolog (PTEN), suppressor of cytokine signaling proteins (SOCS)3 and SOCS4 genes (91). Clearly, formulation into AgNPs played crucial roles to counteract biopharmaceutical limitations of traditional medicinal herb.

In a study, ZnO nanoparticles attenuated oxidative stress, and inflammation to exert protective effects against DN (123). Another study was performed to explore the mechanistic renoprotective benefits of ZnO nanoparticles (124). Results revealed that ZnO nanoparticles to be effective against DN by increasing Nrf2-DNA-binding activity and downregulating thioredoxin-interacting protein (TXNIP) gene expression, which leads to oxidative stress suppression, and impairing inflammatory response by reducing NLRP3 inflammasome activation. ZnO nanoparticles reduce glucose absorption by inhibiting the intestinal α-glucosidase enzyme, improve hepatic glycogenesis by acting on insulin signaling pathways, and enhance glycolysis, thereby improving glucose disposal. They have a potential antiglycation effect by inhibiting the formation of AGEs and preventing changes in protein structure that may occur as a result of covering amino groups in proteins. In a recent study, ZnO-nanoparticles at an oral dose of 10/mg/kg/day for 4 weeks exhibited promise against DN (100). They retained constancy of the glomerular filtration barrier, and restored almost normal renal structure. These ZnO nanoparticles exerted anti-apoptotic, anti-inflammatory, and antioxidant activities to hint at a new therapeutic modality of DN.

Citrate-stabilized Mn_3_O_4_ nanoparticles protect mitochondria, the master regulator of cellular red-ox homeostasis, by scavenging intracellular ROS, inhibiting apoptotic triggers, and preventing antioxidant enzyme loss to alleviate diabetes-associated chronic kidney disease (125). Due to its free radical scavenging activity and high biocompatibility, Mn_3_O_4_ nanoparticles have emerged as one of the most affordable and effective materials for oxidative stress. By avoiding ATP depletion and the opening of the mitochondrial permeability transition pore, Mn_3_O_4_ nanoparticles, exerts protective benefits, by regulating red-ox homeostasis and autocatalysis, Mn_3_O_4_ nanoparticles can lessen oxidative stress, and functionalizing citric acid considerably improves its biocompatibility. The Mn^2+^ ions simultaneously form harmless free radicals that can be effectively managed by biological systems. Adding absence of toxic side effects to it, Mn_3_O_4_ nanoparticles might become superior to oral hypoglycemic drugs currently in use (125).

Metallic nanoparticle posses advantages of stability, easy storability, and sustained availability, thus surpassing the low mechanical properties associated with polymeric nanoparticles. However, metal-based nanoparticles tend to build up in the kidneys since heavy metals are difficult to eliminate via biodegradation. Therefore, searching ways to avoid the unwanted effects by heavy metals has become a focus of current nanoparticle research. There is a possibility that ions produced by metallic nanoparticles may cause injury to the organs involved during excretion. Hence, rigorous research into the toxicity of these nanoparticles to organs during metabolism is required, while quick clearance considerably minimizes the harmful effect of nanomaterials on the system. Nanoparticles larger than 6 nm in size or containing heavy metals could be readily absorbed by the reticuloendothelial system. Because their diameters fall below the threshold necessary for kidney filtration, nanoparticles with small sizes (5.5 nm) are promptly excreted in the urine (126). Some large-sized nanoparticles may be removed by the kidneys following breakdown during prolonged circulation in the body whereby the modified sodium citrate approach might be useful for certain metallic nanoparticles to avoid complexation and enhance circulation time. Again, renal accumulation of metal-based nanoparticles might actually turn up to be a positive indication in terms of therapeutic accumulation at renal sites. The probable toxicity of metallic nanoparticles to the CNS can be minimized by virtue of selective targeting to renal regions (127). Again, to solve solubility issues of some metallic nanoparticles, water-soluble ligands immobilized on nanoparticle surfaces can be utilized to dissolve noble metal nanoparticles in water (128).

#### 5.2.6 Biological nanoparticles

Biomimetic nanomedicine combines the benefits of biomaterials, particularly human cells and pathogens, with the physicochemical features of diverse functional materials. Good biocompatibility, and high accumulation capacity make biological nanoparticles superior candidates among futuristic drug delivery cargos. Viral nanoparticles can integrate the virus’ nucleic acid into the host cell’s nucleic acid with high sensitivity and targeting specificity (129). Influenza A virus nanoparticles have already been used to deliver cinaciguat (degraded by endolysosomes) into renal mesangial cells (40). Cinaciguat from viral nanoparticles can specifically activate the soluble form of guanylate cyclase, which in turn converts guanosine triphosphate to guanosine monophosphate, thereby downregulating glomerular fibrosis. In the experiment under discussion, equivalent efficacy could be observed with merely 10% dose administration of cinaciguat loaded within nanoparticles, compared to free cinaciguat. The dose reduction further aided in reducing biological toxicity, potentially improving patient compliance (40). Like other carriers, Long circulation times tend to improve efficacy and tissue-specific accumulation in case of biological nanoparticles too. Viral nanoparticles bearing negative charge exhibit short half-lives while those with positive surface charge stay in systemic circulation for longer period (130–132). PEGylation has evolved as an effective strategy to minimize biospecific interactions and immunogenicity, and improve circulation time (133). There is a paucity of information on the effectiveness of specifically designed viral nanoparticles *in vivo*. Many studies have demonstrated proof of concept, to underline the strong potential of viral nanoparticles as novel candidates in biomedicinal fields. The next challenge is to better comprehend the outcome and any potential long-term negative effects.

#### 5.2.7 Others

Zuckerman and colleagues (36) have fabricated cyclodextrin-based cationic nanoparticles for delivering siRNA to glomerular basement membrane. These nanoparticles were subsequently coated with an adamantane-PEG conjugate to increase their hydrophilicity, and induce a positive charge. The net positive surface charge has been ascribed to effective drug transport to glomerular basement membrane, and enabled accumulation in the glomeruli. The study has further affirmed that siRNA nanoparticles did not disintegrate in blood; rather, siRNA nanoparticles accumulated and disassembled in the glomerular basement membrane while free siRNA failed to do so. RGDfC (Arg-Gly-Asp-DPhe-Cys)-conjugated QDs have been evaluated for kidney targeting (99). RGDfC-conjugated QDs have been taken up selectively by podocytes that express αvβ3 integrin receptors. A novel mechanism for renal targeting involved altering the size of nanoparticles to meso-scale formulations, as well as altering their opsonization potential (134). Meso-scale nanoparticles for renal proximal tubule targeting have offered significantly enhanced localization in the kidney, as well as remained within kidney for a longer period of time in comparison with other organs. This targeting strategy can be utilized to cater to proximal renal tubule as pressure drop in the nephron, and the large absorptive pressure of the capillaries aid in endocytosis of meso-scale nanoparticles with low opsonization potential into endothelial cells of peritubular capillaries. Human serum albumin peptide fragments can be created as possible renal targeting carriers. Triplotide has been conjugated with PF-A299-585, a peptide fragment of human serum albumin (96). Membranous nephropathy model study has confirmed the usefulness of PF-A299-585 as a carrier for renal targeting, compared to free triplotide. An albumin-bound nanoformulation, nab-paclitaxel has partially ameliorated the microscopic structural changes encountered in the renal cortex during DN, and decreased the immune expression of the fibrogenic mediator TGF-β1 to exert renoprotective effects (95). However, the researchers did not compare the efficacy of native paclitaxel with that of nab-paclitaxel, thus disallowing us to arrive at a definitive conclusion on potential benefits of nanoformulation over native drug. In another study, by interacting with neonatal Fc receptors, albumin nanoparticles specifically entered into renal podocytes, demonstrating the methyl prednisolone-loaded nanoparticles to be specifically absorbed by the podocytes (97). Neonatal Fc receptors -expressing human podocytes displayed an improvement of nearly 36 folds, compared to the uptake in the non-expressing control cells. Co-localization further confirmed that uptake of the uniform, preferable-sized (10 nm) nanoconjugates involved receptor-mediated endocytosis followed by lysosome associated transportation. Another research group reported that nanoparticles of particle size 95 nm were optimum for mesangial accumulation (135). Moreover, compared to free celastrol, celastrol-albumin nanoparticles have exhibited decreased drug accumulation in non-target organs and tissues, thus reducing the systemic toxicity of celastrol. The primary mechanism for the uptake of nanoparticles by mesangial cells was thought to be clathrin-mediated endocytosis. The systemic pressure of the blood flowing into the kidneys and the fenestration of the glomerular capillaries’ endothelium, which allowed the nanoparticles to reach the mesangial cells, were additional factors for the site-specific accumulation of nanoparticles. The cut-off for getting through the filtration barrier is 10 nm, though; as a result, the targeted delivery was successful because nanoparticles were able to localize in the mesangial cells while being unable to cross the barrier (135). To address the obstacles associated with siRNA delivery, cationic dendrimer-templated polymeric nanoplex was conjugated with anionic albumin for delivery of siRNA (48). The approach enhanced serum stability, avoided *in vivo* degradation of free siRNA by RNase and achieved cytosolic delivery of siRNA following endosomal escape. The nanoplex-mediated delivery of siRNA has successfully knocked down targeted gene in both *in vitro* and *in vivo* models of DN. Inhibition of histone deacetylase 4 (HDAC4) by the cargo, in DN subjects makes promise to alleviate podocyte injury, and downregulate HDAC4-STAT1 mediated inflammatory processes. Multifunctional nanoparticles containing metformin have been developed whereby hollow mesoporous silica nanocomposite particles have been doped with trace cerium oxide (for inherent renoprotecvtive activity) were formulated (93). The nanoparticles could mitigate oxidative stress, suppress cellular apoptosis, protect from DN-associated renal injury significantly chiefly by improving renal accumulation over free metformin. Trace cerium oxide has been doped with a multifunctional nanocomposite, to accomplish long-term cyclic scavenging of free radicals in the body. Cerium oxide has a high oxygen storage capacity and improves red-ox characteristics. The hollow silica structure has been etched to retain metformin. Novel photothermal nanoparticles have been prepared that offer outstanding kidney-targeted therapy in presence of near-infrared irradiation for the management of DN (98). In this work, glucose ligand conjugated neutrophil-like cell membrane-coated melanin nanoparticles (Melanin@Glc-NCM) have been synthesized to improve the bioavailability and antidiabetic property of native melanin. Consequently, the formulation could precisely be transported by overexpressed GLUT1 on glomerular mesangial cells. Furthermore the photothermal nanoparticles have restrained hyperproliferation of glomerular mesangial cells after near-infrared irradiation to overcome and/or protect from kidney damage especially during gestational period (98). In this context, a novel attempt to evaluate chitosan-loaded p-coumaric acid nanoparticles hinted at a potential nano-based delivery approach to provide greater nephroprotective effect against DN (104).

## 6 Current challenges and future prospects

DN has emerged as a global impediment associated with DM. For management of DN, renal targeted drug delivery has generated interest among researchers. The use of nanoformulations is an emerging strategy to reach the targeted site efficiently. Polymers, lipids metal-based carriers etc. have been explored for targeted drug delivery to the kidney. The molecular targets of DN are important for site-specific delivery. Nanoformulations are able to overcome constraints like short duration of action, low bioavailability, and dose-related toxic adverse effects, thus hinting at effective strategies for DN management (29). Since it is a progressive nephropathy, early detection and treatment of DN is highly desirable. Therefore, new methods aided with nanoparticulates may offer novel horizon regarding diagnosis of DN.

In the case of polymeric carriers, the renal filtration threshold plays an important role. The renal retention of polymers with molecular weight lower than the renal filtration threshold progresses with post-glomerular processes (10). On the other hand, renal retention of polymers with higher molecular weight generally involves extravasation from the renal vasculature leading to accumulation in the parenchymal tissues (16). From a synergistic therapy perspective, nano-based approaches present unique opportunities to treat complex heterogeneous diseases (136). However, nanocarriers for therapeutic purposes must be safe *in vivo*. Further evaluation in DN is required regarding pharmacokinetics and pharmacodynamics of characteristic proteinuria and altered glomerular filtration rate during pathological conditions (137). Biological nanoparticles e.g. viral carriers seem to be more kidney specific, thus enhancing their bioavailability for renal disorders. However, extensive trials, and *in vitro* and *in vivo* studies are required to evaluate biosafety of such nanocarriers. Compared to other modalities, the aim of nanoplatform-based drug delivery is to deliver drugs precisely to renal sites to interact with multiple pathogenic pathways. The application of multifunctional targeted nanoparticles in DN diagnosis is a development trend, whereby successful clinical translation would require long-term efficacy and safety assessments. Certain carriers have been designed with ligands for efficient, site-specific delivery of diagnostic or therapeutic agents into the renal region.

The propensity of the kidney to expel drugs out of the system makes developing a platform for drug administration within the kidneys for the treatment of DN highly challenging for formulation scientists. Though treatment modalities involving mRNA seem to be a promising approach, mRNAs face obstacles such as fast nuclease destruction, macrophage phagocytosis removal, and renal filtration clearance. Nanoencapsulation can be an effective approach to solve major issues. However, safety and adjuvancity of mRNA-nanocarriers is still a matter of concern (138). Since the ground-breaking mRNA COVID-19 vaccines have already shown the great potential of mRNA nanomedicines, we anticipate that ongoing innovation will result in novel and extremely effective mRNA-based therapeutics for other diseases (including DN) too. The vast number of nanoformulations at preclinical and clinical stages of evaluation make it clear that the field of nanomedicines is destined to gain a sizable market share in the near future. In terms of DN, specialized biophysical properties of nanomedicine-based techniques enable improved contact with renal cells to promote improved retention as well as improved cellular uptake inside the basal membrane epithelial cells, podocytes, mesangial cells, and proximal tubule cells. New insights into the pathophysiology of DN and knowledge of the structure and function of the kidney serve as effective tools for optimizing medication delivery systems for DN research. It’s time for nephrology specialists and nanotechnological research teams to work together to create energizing nanomedicine strategies for the clinical translation of specific targeting and therapy of DN.

## 7 Discussions

**Figure 5:**
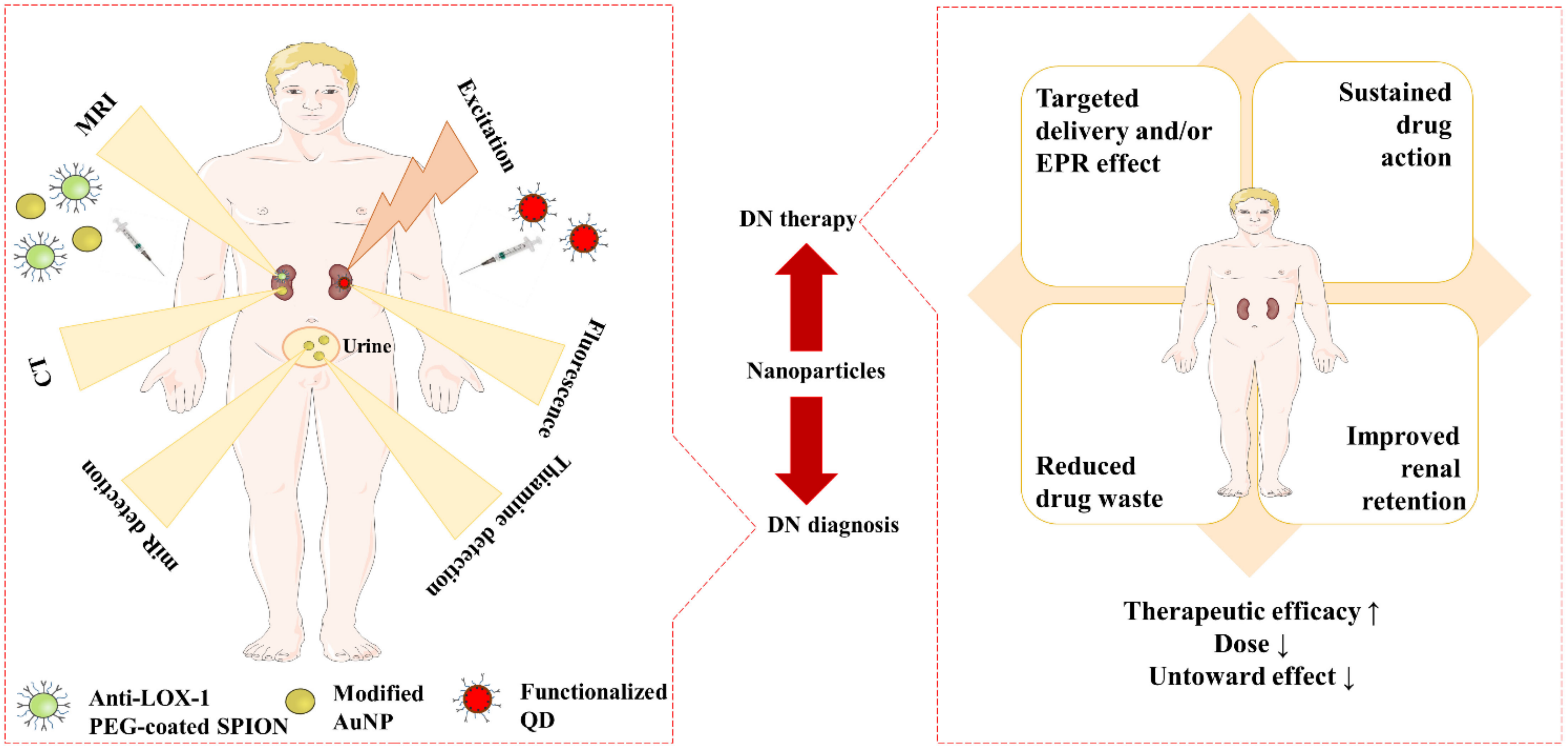
Advantageous roles of nanoformulations in DN diagnosis, and DN therapy. ‘↑’ indicates increase, ‘↓’ indicates decrease. AuNP, Gold nanoparticle; DN, Diabetic nephropathy; EPR, Enhanced permeability and retention; PEG, Polyethylene glycol; QD, Quantum dot; SPION, Super paramagnetic iron oxide nanoparticle.

DN has become a significant issue in both type 1 and type 2 DM globally. The course of DN may be potentially slowed down and/or halted by treating underlying DM because all of the processes are linked to underlying hyperglycemia. Oral hypoglycemics, presently in use often aggravate kidney damage (139). By eliminating undesirable traits of potential therapeutic agents, including short duration of action, low bioavailability, and toxic adverse effects, nanoparticles can offer effective therapeutic methods for the treatment of DN. Moreover, early detection and treatment of DN are particularly desirable due to the fact that it is a progressive disorder. Novel methods for the diagnosis of DN might also be provided by nanoparticle-based techniques. **Figure 5** explains roles of different nanoformulations regarding diagnosis and treatment of DN.

Polymeric nanoparticles may be useful in overcoming the renal filtration threshold due to their low molecular weight, which permits them to be filtered through the kidneys and be kept in the kidneys via post-glomerular processes. Because of their extended retention time, ultrafine size, and capacity to release medications for prolonged periods of time, chitosan nanoparticles have become highly desirable as oral delivery vehicles. As was previously mentioned, research on cutting-edge nano-drug delivery systems is already on the way, but more study is required to evaluate the typical proteinuria in pathological conditions, as well as changes in glomerular filtration rate in pathological conditions, and to design an optimal drug delivery system for DN. The tendency of biological nanoparticles to be more kidney-specific leads to an increase in their efficacy, examples include those that imitate the sequence recognition approach of the influenza A virus (40). However, extensive trials, as well as *in vitro* and *in vivo* research, are still needed to assess the toxicity and biosafety of viral nanoparticles. Though there exist chamces of AgNPs-mediated inflammation and cell damage in the kidneys, AgNPs still represent a promising nanoformulation for diagnostic applications. The main site for accumulatrion is liver (not kidney), which can again possibly be minimized via citrate modification (140). Moreover, silver can be cleared from most of the organs within 8 weeks post-dosing (141). Even, evidence suggests that acutely ingested AgNPs, irrespective of size or coating are well-tolerated (142). On this note, in a study, no harmful effects in liver and kidney were observed efter 90 days exposure to AgNPs (143). Another study reported a dose upto 10 mg/kg of AgNPs to rats to be safe while higher doses exerted toxic effects (144). Apart from dose, particle surface area, surface chemistry, and meticulous, accurate characterization of particle size and morphologic properties continue to be key issues regarding efficacy and toxicity, particularly in the physiological environment (145).

The presence of several targeting windows within the sick nephron, such as size cutoff, presence of charge-bearing components, and availability of particular receptors, can be used to construct functional nanoparticles for targeted therapeutic and/or diagnostic reasons (**Table 2**). Glucose or other sugar base ligands may be employed to target GLUT1 on glomerular mesangial cells for effective and targeted drug delivery in DN, resulting in improved bioavailability of several drugs (68, 98, 109, 139). A major trend that will require long-term efficacy and safety assessments, is the use of multifunctional targeted nanoparticles in DN diagnosis. Although the practicality of utilizing nanoparticles in kidney-targeted distribution has been established using excellent size-control and mesangial filtration, the nanoparticles would be excreted in urine quickly if they are not internalized swiftly by renal cells. Furthermore, most renal cells in DN patients, particularly renal tubular cells, exhibit cellular uptake dysfunctions of glucose, protein, and mineral salt, as well as nanoparticles, impeding improvements in the therapeutic efficacy of nanoparticles (146). Thus, efforts to overcome the challenges of inadequate cellular absorption by renal cells and fast urinary excretion of nanoparticles in DN treatment remain necessary.

**Table 2:** Targeting strategies of nanoformulations to renal cells.

S No.	Targeting types	Targeting mechanisms/strategies	Outcomes	References
1	Active	VCAM-1 and E-selectin specific antibodies	Selective delivery of siRNA to inflamed cells with amphiphile-modified liposomes	(62)
2	Active	Anti-VCAM-1 antibody	Targeted delivery to TNF-α activated podocytes	(63)
3	Passive	Ultrasound-mediated targeting	Directed delivery to target region	(64, 66)
4	Active	Kidney-targeted peptide	Kidney-specific distribution	(67)
5	Active	Interaction of glucose-ligand with GLUT1 receptor	Preferential renal distribution	(68)
6	Active	Megalin-mediated uptake of polymeric nanoparticles	Kidney-specific delivery of loaded cargo	(76, 80)
7	Active	Mannose or transferrin targeted delivery	Mesangium-specific delivery of siRNA	(78)
8	Passive	Brij-functionalized polymeric nanocarriers	Improved permeability	(87)
9	Passive	pH and glucose responsive delivery	Reduction of blood glucose to ameliorate DN	(88)
10	Passive	Size-dependent retention in mesangium	Amelioration of mesangial proliferative glomerulonephritis	(94)
11	Active	Peptide fragments of human serum albumin	Improved renal delivery of triplotide	(96)
12	Active	Neonatal Fc receptors targeted delivery	Specific absorption by podocytes	(98)
13	Passive	Photo-thermal nanoparticles	Improved distribution to mesangia0l cells	(99)
14	Passive and active	Anti-LOX-1, PEG-coated SPIONs	Detection, Characterization, and monitoring of early DN	(51)
15	Active	Anti-TLR4 and anti-AR antibody-tagged QDs	Fast, accurate detection	(55)

Clinically, microbubbles are utilized as ultrasound contrast agents, and investigated as an ultrasound therapy mediator. To enhance the theranostic effect, microbubbles can be attached to the nanoparticles with the intention of targeting, permeation, imaging, and enhancing acoustic response under ultrasound waves. The particle size of microbubbles, like that of nanoparticles, and surface display of targeting ligands, are significant aspect for drug delivery to the kidney (64, 66). Researchers are still working on the microbubble therapy technology for drug delivery to the kidney as an emerging delivery mechanism.

Although biodegradable polymers like PLA are widely thought to be benign, their immunotoxicological potential should be carefully assessed as smaller sized particles tend to cause more damage to normal cells (147). Potential toxicological hazards of metallic nanoparticles also need to be carefully addressed before rampant clinical utilization. One of the most difficult problems in the clinical use of nano-biomedicine is how to deal with the biotoxicity of bio-nano drug-loaded particles and their breakdown products, which requires more investigation.

Poly(vinylpyrrolidone-co-dimethyl maleic acid) has been shown to have a high accumulation potential in the kidney and can be conjugated with superoxide dismutase for the treatment of renal illnesses. The molecular weight and charge of the copolymer effect its distribution. Copolymer with a molecular weight of 6-8 kDa accumulates in the kidneys; the stronger the negative charge, the longer it takes for it to be eliminated from the kidney (41). The copolymer poly(vinylpyrrolidone-co-dimethyl maleic anhydride) has been reported to be quickly removed from circulation, while accumulating in proximal tubular cells (148). For the treatment of renal disease the copolymer was conjugated with superoxide dismutase to suppress ROS production and the extracellular signal-regulated kinase (ERK) signaling pathway in DN (148). Poly-l-glutamic acid is a renal polymeric drug carrier that is selective for renal protective medicines with advantages like high drug-loading, non-immunogenicity, biodegradability, and biocompatibility. The selective absorption of fluorescence-tagged poly-l-glutamic acid of molecular weight 41 kDa by kidney epithelial cells was reported in a study (42). While the renal retention of higher molecular-weight polymers typically involves extravasation from the renal vasculature and deposition in the parenchymal tissues, the renal retention of polymers with a molecular weight lower than the renal filtration threshold involves active reuptake of polymers by the proximal tubules. Hence, there exists strong possibility of renal-targeted delivery of polymeric nanoparticles utilizing such copolymers. In addition, antibody and peptide ligands showing promise in renal targeting can further improve the site-specificity (16).

A randomized, placebo-controlled double-blind clinical trial evaluated efficacy of curcumin-loaded nanoformulations prepared using polysorbate 80 (149). Encapsulation of curcumin into nanoformulation enhanced its bioavailability, resulting in increased antioxidant activity of curcumin along with better therapeutic response against DM. The safety and tolerability of the nanoformulation was also ascertained (80 mg nanocurcumin for 8 weeks) hinting at possibility of commercialization. In another relevant trial, significant improvements were observed regarding DM parameters in patients, using curcumin nanomicelles at a dose of 80 mg/day for 3 months (150). The therapeutic effects found in this study are thought to be attributable to the improved bioavailability of curcumin when delivered as nano-micelles. Future studies should concentrate on the production processes and thorough clinical studies of nanoformulations leading to their quick availability for utilization. A multidisciplinary strategy combined with clinical and ethical considerations is needed to integrate nanotechnology into ordinary clinical practice. Additionally, the synergistic effect of combining flavonoid nanoformulations with conventional medication therapy for diabetes could be examined. However, successful clinical translation still lies in the womb of the future.

## 8 Conclusion

Because of the kidney’s propensity to expel medications from the body, targeting the kidneys in DN is a huge challenge. Since DN therapy necessitates high renal drug concentrations, the development of concentration-dependent targeted drug delivery devices is required. The development of nanotheranostic platforms improves treatment efficacy and safety while also providing new precision therapy measures for patients with DN. This field of nanomedicine is quickly expanding, as indicated by the various nanoparticles that have already been produced. Nanomedicine-based techniques with specific biophysical properties could result in highly regulated nanocarriers for kidney targeting. Nanoparticles would be able to connect with renal cells for an extended period of time, improving retention and cellular uptake in numerous cell types. However, additional significant efforts have to be initiated by the researchers to develop clinically effective targeted nanoformulations containing diagnostic and/or therapeutic moieties. Future research is anticipated to progressively concentrate on drug delivery systems based on nanoparticles, with the molecular targets of DN being essential for site-specific targeted delivery.

## Author contributions

SD, RK, SJ and NJ contributed to the conceptualization of the manuscript. PP, LC, TD, VP, and UP designed the first draft of the manuscript. SJ, NJ, RK, and PC edited and corrected the manuscript. The final correction and editing were done by SD, PC, and RK. All figures were formulated by SD All authors contributed to the article and approved the submitted version.
